# Advanced therapies in Parkinson’s disease: an individualized approach to their indication

**DOI:** 10.1007/s00702-024-02773-3

**Published:** 2024-04-13

**Authors:** Nils Schröter, Bastian E. A. Sajonz, Wolfgang H. Jost, Michel Rijntjes, Volker A. Coenen, Sergiu Groppa

**Affiliations:** 1 Department of Neurology and Clinical Neuroscience, Medical Center, Faculty of Medicine, University of Freiburg, Freiburg, Germany; 2 Department of Stereotactic and Functional Neurosurgery, Medical Center, Faculty of Medicine, University of Freiburg, Freiburg, Germany; 3 Parkinson-Klinik Ortenau, Wolfach, Germany; 4 Center of Deep Brain Stimulation, Medical Center, Faculty of Medicine, University of Freiburg, Freiburg, Germany; 5 Section of Movement Disorders and Neurostimulation, Department of Neurology, University Medical Center of the Johannes Gutenberg-University Mainz, Mainz, Germany

**Keywords:** Parkinson’s disease, LCIG, CSAI, DBS, Device aided therapies

## Abstract

Device aided therapies (DAT) comprising the intrajejunal administration of levodopa/carbidopa intestinal gel (LCIG) and levodopa/carbidopa/entacapone intestinal gel (LECIG), the continuous subcutaneous application of foslevodopa/foscarbidopa or apomorphine infusion (CSAI) and deep brain stimulation (DBS) are used to treat Parkinson’s disease with insufficient symptom alleviation under intensified pharmacotherapy. These DAT significantly differ in their efficacy profiles, indication, invasiveness, contraindications, and potential side effects. Usually, the evaluation of all these procedures is conducted simultaneously at the same point in time. However, as disease progression and symptom burden is extremely heterogeneous, clinical experience shows that patients reach the individual milestones for a certain therapy at different points in their disease course. Therefore, advocating for an individualized therapy evaluation for each DAT, requiring an ongoing evaluation. This necessitates that, during each consultation, the current symptomatology should be analyzed, and the potential suitability for a DAT be assessed. This work represents a critical interdisciplinary appraisal of these therapies in terms of their individual profiles and compares these DAT regarding contraindications, periprocedural considerations as well as their efficacy regarding motor- and non-motor deficits, supporting a personalized approach.

## Background

Parkinson’s disease results in motor and non-motor symptoms that collectively contribute to the disease burden for individual patients (Martinez-Martin et al. 2011; Armstrong and Okun 2020; Bloem et al. 2021). After an initially favorable response to oral pharmacotherapy, patients develop motor and non-motor fluctuations that significantly worsen the quality of life and make further treatment more difficult (Storch et al. 2013; Hechtner et al. 2014). These fluctuations are in part associated with dysphagia or impaired gastrointestinal transit, leading to fluctuating levodopa plasma levels (Hardoff et al. 2001; Pflug et al. 2018). To ameliorate these fluctuations, patients receive complex medication plans with a higher frequency of intake times as the disease advances. This contributes to non-compliance with medication that is correlated with an elevated frequency of motor and non-motor complications, additionally incurring significantly higher costs for the healthcare system (Olanow et al. 2006; Davis et al. 2010).

The term “advanced” Parkinson’s disease is increasingly being used for a phase with *refractory* symptoms and fluctuations (Luquin et al. 2017). At this point in disease progression, advanced device aided therapy (DAT) methods are typically of need. DAT can be categorized into three different forms of administration. Currently, there are two different applications that administer their agents directly intrajejunally: levodopa/carbidopa intestinal gel (LCIG) and the more recent levodopa/carbidopa/entacapone intestinal gel (LECIG). Similarly, there are two approved application forms that administer their compounds subcutaneously (s.c.), namely apomorphine and foslevodopa/foscarbidopa. As a fundamentally different, non-pharmacological mechanism, deep brain stimulation (DBS) is also available (Timpka et al. 2017; Antonini et al. 2018, 2023).

Choosing the appropriate therapy and timing for the individual patient poses a challenge. Depending on the clinical scenario, a patient may undergo multiple procedures consecutively, such as apomorphine as a bridging therapy for later DBS or pump therapy. It is also possible to combine therapies (Pürner et al. 2023). To assist in the decision-making process, an international guideline was published in 2022 (Deuschl et al. 2022). However, as the guideline by its nature was primarily based on high quality randomized controlled studies, the information of smaller studies and personal expertise are lacking. In a second publication, more specific recommendations for individual patient groups were derived based on this guideline; however, this work does not include LECIG and foslevodopa/foscarbidopa (Brinker et al. 2023).

Other crucial factors affecting a patient’s access to continuous therapy are economic considerations, as these therapies come with increased costs. Additionally, regional availability and patient-specific factors such as gender, social and care-giver issues play an important role (Mathur and Stamford 2021). As part of a large international observational study only 44% of eligible patients with PD patients received a DAT, while those who did receive DAT displayed significantly fewer motor deficits and fewer impairment in everyday activities (Fasano et al. 2019).

The reasons for the lack of indication and realization are complex and can probably be found in the inadequate provision of information to patients and caregivers, insufficient incentives for continuous procedures in the outpatient sector, and the simultaneous challenges posed by increasing cost pressures in the healthcare system.

Furthermore, many experts do now believe that the above-mentioned DATs, despite their different uses and indications, complement purely oral therapy at exactly the same time (“advanced PD”) and are therefore equivalent to each other. This publication questions this strategy, proposes an individualized approach for individual patients and aims to provide an objective summary of current research on the efficacy of continuous therapy methods for Parkinson’s disease, comparing and contrasting them to create a more nuanced understanding for personalized therapy decisions. The authors propose that each therapy may be applicable at varying stages of the disease’s progression, necessitating specific differential therapeutic considerations.

## Continuous intra-jejunal administration of levodopa (LCIG/LECIG)

Levodopa is administered in the form of a gel through a percutaneous endoscopic jejunal tube (PEJ) in both LCIG and LECIG. This allows for the medication to bypass the act of swallowing and stomach passage, resulting in more consistent and effective levels of the drug (Olanow et al. 2006). One positive aspect of LCIG therapy is the ability to test its efficacy via a nasojejunal tube before PEJ insertion. There is continuing effort involved in changing cartridges and connecting/disconnecting the pump.

As early as 2005, a randomized, controlled multicenter study demonstrated the superiority of LCIG over the best oral medication in terms of reducing motor deficits and improving quality of life in a cohort of patients suffering from advanced PD (Nyholm et al. 2005). Subsequently, the effectiveness was also confirmed in larger randomized studies and a decrease in dyskinesia affecting patients was also observed (Olanow et al. 2014).

In addition to its impact on motor deficits, this treatment also showed a significant decrease in non-motor deficits, specifically in the areas of cardiovascular symptoms, gastrointestinal symptoms, sleep, fatigue, mood cognition, attention and memory as well as dyskinesia. These findings were supported by multiple further studies (Antonini et al. 2017; Cruse et al. 2018; Freire-Alvarez et al. 2021). Of note, a multinational observational study found that the percentage of patients treated with LCIG monotherapy increased from 15 to 32% (Fasano et al. 2021). This can be viewed positively both for compliance and for reducing the risk of drug interactions in the context of polypharmacy in older individuals.

Another recent development is the extension of the approved period for application from 16 to 24 h. This is especially relevant for patients with sleep-related deficits such as OFF-associated dystonia and reduced quality of sleep (Busk and Nyholm 2012).

A few years ago, LECIG was approved as second method for intrajejunal levodopa administration. In addition to levodopa/carbidopa, LECIG contains the COMT inhibitor entacapone, leading to more consistent elevated plasma levels of levodopa (Senek et al. 2017). This allows for a reduction in the total volume of the gel per cartridge, enabling the use of a smaller and quieter pump compared to LCIG, which patients considered beneficial (Öthman et al. 2021).

Both LCIG and LECIG share similar adverse drug reaction (ADRs) spectra. The reported frequency of ADRs ranges between 10% and 90% depending on individual studies. In addition to probe dislocations and local wound healing disorders and infections, abdominal pain and polyneuropathies are also observed (Antonini et al. 2017; Grund et al. 2023). The duration of the disease before initiating LCIG therapy ranged from 12.5 (± 5) to 14 (± 4) years (Fernandez et al. 2015; Martinez-Martin et al. 2015).

## Continuous application of subcutaneous foslevodopa/foscarbidopa

Only recently, foslevodopa/foscarbidopa was introduced to the market. The incorporation of this phosphate prodrug has facilitated a more than hundredfold increase in the solubility (Rosebraugh et al. 2021a, b). This alteration enables the subcutaneous administration of foslevodopa/foscarbidopa, providing a practical and minimally invasive route for therapeutic delivery. It can be given over a 24 h period.

Due to its only recent availability, there are limited data, for the differential therapy of specific non-motor deficits. However, in a comprehensive Phase 3 study, not only an increase in ON-time and a reduction in OFF-time were observed, but also an improvement in sleep quality and overall quality of life. Commonly reported side effects included infusion site erythema, nodules, and cellulitis, as well as pain and visual hallucinations (Soileau et al. 2022; Aldred et al. 2023). Additionally, a major limitation of foslevodopa/foscarbidopa is the lack of long-term data on efficacy and tolerability, furthermore, data on long-term efficacy as monotherapy is currently lacking.

In summary, initial data shows a benefit of foslevodopa/foscarbidopa in Parkinson’s disease with motor fluctuations. However, further research is warranted to establish a more definitive understanding of the therapeutic benefits and potential limitations associated with the use of foslevodopa/foscarbidopa in clinical practice.

## Continuous application of subcutaneous apomorphine (CSAI)

Apomorphine, a dopamine D1- and D2-receptor agonist, has been employed in Parkinson’s therapy for many years. Due to its significant first-pass effect, the administration is restricted to parenteral routes. That is why subcutaneous application is therefore employed, either intermittently via pen for addressing delayed-on phenomena or sudden-offs, or continuously via pump for managing fluctuations (Trenkwalder et al. 2015). More recently, sublingual administration of apomorphine was introduced (Olanow et al. 2020).

In analogy to LCIG, bypassing the gastrointestinal tract by continuous subcutaneous apomorphine infusion (CSAI), allows for continuous drug administration and absorption, resulting in more stable drug plasma levels. In a large randomized controlled study, this approach demonstrated a significant reduction in OFF time and increase in ON time without troublesome dyskinesias for individuals with Parkinson’s disease (Katzenschlager et al. 2018).

In addition to addressing motor deficits, several smaller open-label studies suggest an improvement in non-motor deficits with the use of apomorphine. Particularly, sleep quality has shown enhancement in multiple studies, attributed partly to a positive influence on concomitant Restless Legs Syndrome. Furthermore, a positive impact on apathy and depression has been documented, along with a reduction in nocturia (Todorova and Chaudhuri 2013; Meira et al. 2021; Kukkle et al. 2023).

One limitation of CSAI is that it is not sufficiently effective as a monotherapy but requires the administration of additional dopaminergic medication. Typical side effects include the occurrence of skin nodules at the infusion site, observed in nearly half of the patients, as well as nausea and somnolence, each reported in approximately one-fifth of the patients. Additionally, new impulse-control disorders (ICDs) have been observed in CSAI (Martinez-Martin et al. 2015). However, a small case series suggested that pre-existing ICDs can either partially improve or completely cease upon initiation of therapy (Todorova et al. 2015). Nevertheless, based on clinical experience, the authors are rather reluctant of the use of CSAI in patients with ICDs and see this as the DAT of last resort in this population. Contraindications for the use of apomorphine include the presence of therapy-refractory orthostatic hypotension, severe cognitive deficits, and therapy-refractory psychosis. However, in cases where mild visual hallucinations are present, and the patient can clearly distance themselves, careful consideration on a case-by-case basis may permit the use of CSAI (Katzenschlager et al. 2018). This is supported by a case series of patients with severe visual hallucinations that reported no deterioration after treatment initiation with CSAI (Laar et al. 2010).

## Deep brain stimulation (DBS)

Stereotactic lesional therapy beginning in 1947 set the ground for modulation of specific neuronal networks to treat symptoms of movement disorders amongst them PD (Cif and Hariz 2017). In contrast to lesional therapies, DBS can be precisely adapted, allowing for delicate targets to be approached like the subthalamic nucleus (STN) in order to treat rigidity, akinesia, and tremor in PD and thereby reducing L-dopa equivalent dose and attenuating motor fluctuations (Krack et al. 1998; Limousin et al. 1998). These results have been replicated in a variety of studies spanning up to five years after surgery (Kleiner-Fisman et al. 2006) and more recent reports have been able to show that a therapeutic effect is maintained well beyond 10 years after surgery despite natural ongoing disease progression (Bove et al. 2021; Hacker et al. 2023). Superiority of STN DBS over best medical treatment has been demonstrated in suitable patients (Deuschl et al. 2006).

STN DBS was initially applied as last line therapy when all other treatment options were exhausted (Kleiner-Fisman et al. 2006). The EARLYSTIM trial opened up a new perspective for STN DBS as an effective treatment for early motor fluctuations (Schuepbach et al. 2013). However, the compelling question, whether STN DBS should generally be applied in younger patients with shorter disease duration remains difficult to answer as controlled studies addressing this issue are lacking. Reviewing several prospective and retrospective studies, Geraedts et al. did not find any clinical or sociodemographic factor that consistently predicts the quality of life after STN DBS (Geraedts et al. 2020). In contrast, a review article by Mahlknecht et al. concludes that STN DBS may delay some late-stage disability and slightly prolong survival (Mahlknecht et al. 2022). Together these findings underline that the optimal time point for DBS surgery is not tied to a specific age or disease duration, but should be determined individually based on constant evaluation.

DBS surgery should not be postponed unnecessarily in suitable patients, as this may increase perioperative morbidity or even deprive some patients of the benefits of DBS therapy at all, if they fail to meet suitability criteria later on.

The question of the optimal DBS target is not the topic of this manuscript. With the STN we only covered the most extensively studied DBS target for PD so far; however, different targets like Globus pallidus internus (GPi) and ventral intermediate nucleus of the thalamus (Vim) exist and should be considered individually to match patient- and disease-specific factors (Honey et al. 2017). Comparing the medication ON state, GPi and STN DBS achieved equal outcomes for motor performance and quality of life in randomized trials up to 36 months after surgery (Follett et al. 2010; Weaver et al. 2012; Odekerken et al. 2013, 2016). However, reduction of dopaminergic medication (and its side effects) point to the STN as a target, while dystonic features, cognitive impairments, psychiatric comorbidities point to the GPi (Honey et al. 2017).

Vim-DBS is the traditional approach for therapy-resistant tremor (Benabid et al. 1991), but in the context of PD STN DBS can also successfully alleviate tremor (Krack et al. 1998). Hence, additional criteria should be taken into account when considering DBS in the Vim, such as the extent of other cardinal Parkinson’s symptoms in addition to tremor and possible contraindications for the STN or GPi target. Furthermore, additional DBS in the Vim can also be considered if existing stimulation in the STN or GPi fails to satisfactorily reduce tremor (Honey et al. 2017).

In general there is less insight into how DBS affects non-motor symptoms (Kurtis et al. 2017). Recent studies suggest general improvement of non-motor symptoms by STN DBS (Jost et al. 2020) with a relevant impact of patient- and disease-specific factors on non-motor symptoms outcome (Jost et al. 2021) as well as location of active electrodes contacts in the STN (Dafsari et al. 2018). Beneficial effects on non-motor symptoms have also been reported for GPi-DBS with a different pattern across non-motor domains. In patients with impulse-control and related disorders (ICBDs), DBS, especially of the STN led to symptom alleviation in several prospective studies, potentially by the decrease in the dopaminergic medication, for review see (Debove et al. 2024).

Important new developments have been introduced in recent years. An important milestone was the introduction of directional stimulation, which (1) allows the current to be delivered to specific pathways/regions that are responsible for the effects and (2) minimizes the spread of electrical stimulation to structures that cause side effects (Schnitzler et al. 2022). In clinical practice, this potentially means fewer patients with dysarthria due to current spreading into the pyramidal tract, fewer hypomanic symptoms in patients with STN DBS for stimulation of limbic STN areas (Reker et al. 2016).

Another important step was the development of image-guided programming, somewhat hand in hand with the introduction of directional stimulation. This allows robust selection of the dorsal to ventral contact level for the stimulation site, but also reduces the number of selections for the active contacts of directional electrode leads (Lange et al. 2021; Rolland et al. 2024).

Several recent studies have investigated the potential of asleep versus awake DBS surgery with MER recordings and intraoperative testing (Qian et al. 2023). Although the general results show comparability of postoperative UPDRS scores, patients implanted awake with standardised intraoperative testing/macrostimulation show fewer postoperative stimulation-related side effects. It is also our experience that in at least one or two out of five patients, deviation from the central trajectory during intraoperative testing is necessary to achieve the clinical effects or to reduce the lower threshold side effects.

Emerging closed-loop approaches have been introduced for PD and tremors and will be emergently designed to adapt stimulation parameters to electrophysiological surrogates of disease symptoms and states. CL-DBS paves in our view the way for adaptive personalised DBS protocols. Future studies should address however the perspectives of CL technology and its opportunities and potential pitfalls for clinical use (Bouthour et al. 2019; Groppa et al. 2023).

In recent years, there has been growing evidence that genetics also contribute to the effectiveness and potential side-effects of DBS. Currently, patients with a GBA mutation are particularly in focus, as retrospective studies suggest a correlation between a significantly pronounced decline in cognitive function under DBS and the presence of this mutation (Pal et al. 2022; Avenali et al. 2024). On the other hand, a large meta-analysis confirmed improved motor outcomes across patients with monogenetic Parkinson’s disease (Artusi et al. 2019).In summary, the relevance of genetic variants remains unclear; however, there are indications that they will increasingly play a role in the selection of suitable patients in the future.

## Comparison of device aided therapies

Various meta-analyses find important effects of DAT in improving quality of life for PD patients and caregivers (Nijhuis et al. 2021; Rajan et al. 2022). To date there are no data from large clinical randomized head-to head comparison of LCIG/LECIG, foslevodopa/foscarbidopa, CSAI and DBS, as such that comparisons about their effectiveness and long-term outcomes should be carried out with appropriate caution. All DAT have their own strengths and weaknesses that must be taken into account when determining the indication and advising patients (Timpka et al. 2017).

Before therapy induction, LCIG/LECIG allows prior testing of the effectiveness of the procedure via a nasojejunal tube. This can be performed prior to PEJ. One advantage of foslevodopa/foscarbidopa and CSAI is that they do not require surgery, and the therapeutic effect can be easily assessed. In the case of CSAI, subcutaneous administration using a pen is available. In DBS, the likely response to therapy can be anticipated indirectly through a levodopa challenge-test. With regard to the age at the start of therapy, there is no limit for LCIG/LECIG and CSAI, while an age limit for DBS is often discussed, as the perioperative risks are increased with progressed age and existing cognitive impairment. The presence of dementia is a clear contraindication for CSAI and DBS. The situation is analogous in the case of severe depression. If improvement of depression cannot be achieved, this is also a contraindication for DBS, but CSAI and LCIG/LECIG can possibly be applied. The presence of severe orthostatic hypotension as well as severe psychosis are contraindications for CSAI. Frequent falls with injuries might be a contraindication for DBS. Patients who want to maintain physical activities might be hindered by carrying an external device.

LCIG and CSAI require some care with changing cartridges and handling the pump. Therefore, in most cases, daily support in the home environment from relatives or nursing services is necessary. Although regular outpatient checks are required for DBS, usually with a tertiary referral center, no further support in everyday life is typically required to ensure the effectiveness of the therapy. To use LCIG/LECIG or CSAI, the pump must be carried in a bag that produces a certain noise level (especially with LCIG). In contrast, the DBS does not require any externally visible or audible technology other than the charger (for rechargeable systems).

With regard to the therapeutic response, a major strength of DBS over LCIG and CSAI is the treatment of tremor that cannot adequately be treated with levodopa as well as a positive modulation of dyskinesias. While a good response is usually observed with DBS, suboptimal improvement can often be expected with LCIG or CSAI. As with DBS, foslevodopa/foscarbidopa has been approved for continuous use over 24 h, so that therapy can be given without discontinuation, which is a relevant advantage over CSAI in particular.

It is therefore clear that LCIG/LECIG, CSAI and DBS differ significantly in terms of handling, the profile of side effects and contraindications, and their strengths in symptom control. Although these methods are often viewed as competitors, they have different strengths, weaknesses and different target symptoms, therefore aiming at distinct patient groups at distinct points of their individual symptom evolution.

This is also supported by several recent studies, which have shown that patients also benefit from a combination or sequential application of various intensified therapies. Several retrospective case series show that both the combination and the sequential use of intensified therapeutic procedures can lead to significant clinical improvement (Sesar et al. 2019; Georgiev et al. 2022; Pürner et al. 2023). This is supported by a small prospective case series that showed a significant decrease in motor impairment with the addition of LCIG in patients with DBS and therapy-refractory symptoms (Regidor et al. 2017).

The mere occurrence of motor fluctuations does not necessarily imply a decrease in quality of life, especially in the initial stages (Marras et al. 2004). However, if a loss of quality of life becomes apparent, a DAT should be considered. The 5-2-1 rule was formulated by an expert panel to advise on the optimal timing for consulting a movement disorders specialist for an evaluation of DAT in PD. This rule postulates that, despite a minimum of five daily medication administrations, there should be at least two hours of OFF time and one hour of dyskinesia per day before DAT is considered (Regidor et al. 2017). For the next step of assessing these patients, the MANAGE-PD tool was developed. This validated online tool is freely available, easy to use and aims to identify individuals with advanced Parkinson’s disease who may be experiencing inadequate control of both motor and non-motor deficits (Antonini et al. 2021; Südmeyer et al. 2023). The utilization of such tools aligns with the goal of reaching a broader population and improving the identification of patients who could benefit from advanced therapeutic interventions.

Considering that a delayed implementation of DAT is not only linked to an unnecessary loss of quality of life but also correlates with an elevated peri-interventional risk, we advocate for the early and recurrent assessment of DAT. In our perspective, such assessments should be conducted whenever a patient experiences limitations in social life or endures a diminished quality of life.

## A patient centered approach

**Fig. 1:**
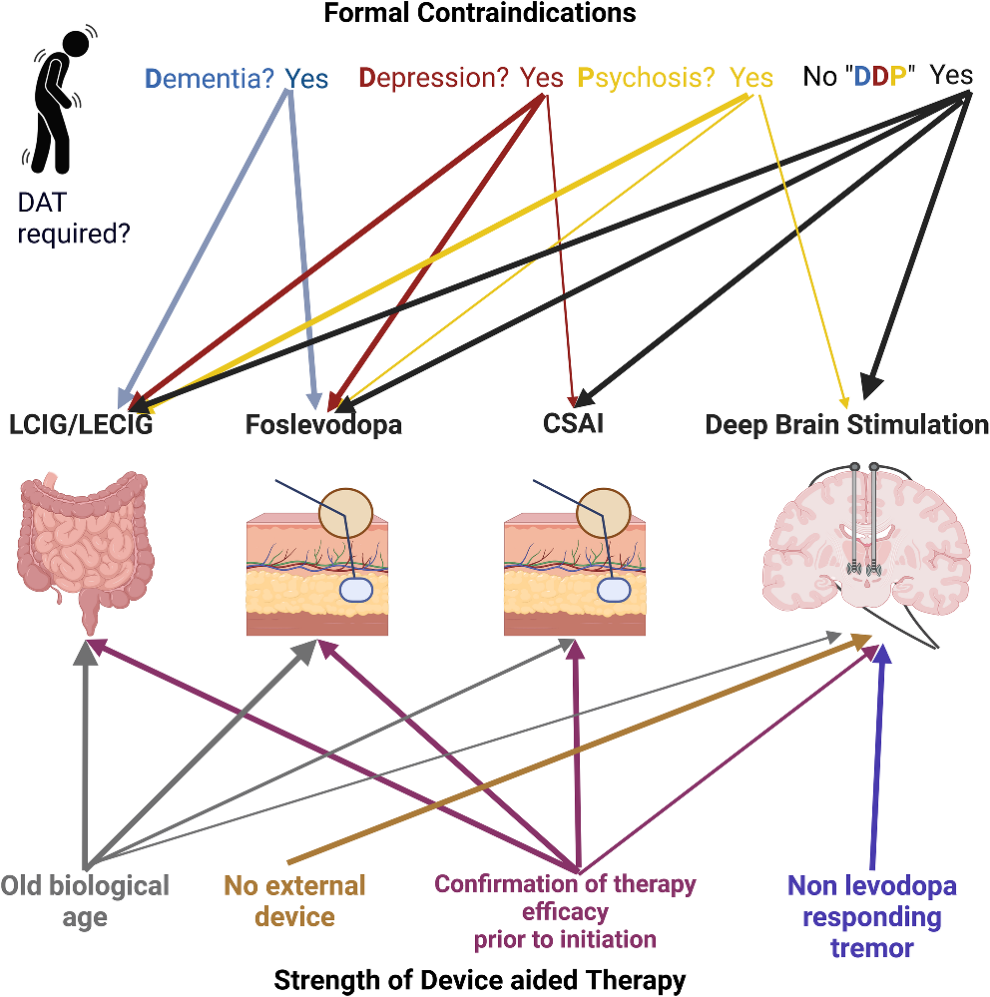
This figure visualizes contraindications and strength of current device aided therapies (DAT) in Parkinson`s disease. The stronger lines visualize that a certain DAT is particularly suitable in the presence of this disease or symptom. The absence of a line means that this procedure should not be used if this disease or symptom is present. DAT, device aided therapy; DDP, Dementia, Depression, Psychosis. LCIG, levodopa/carbidopa intestinal gel; LECIG levodopa/carbidopa/entacapone intestinal gel; CSAI, continuous subcutaneous application of apomorphine; foslevodopa, foslevodopa/foscarbidopa. Created with BioRender.com

Several recommendations for the treatment of individual patients can be derived from this. In patients without relevant medical or psychiatric comorbidities, as well as cognitive deficits, all DAT are available. Due to the simpler everyday handling and the absence of the need to visibly carry a pump, these patients should be early candidates for DBS. If this is not desired by the patient, e.g. due to concerns about brain surgery, foslevodopa/foscarbidopa and CSAI could be considered as a second option, as both methods involve s.c. administration, allowing for the avoidance of placing a PEJ. In the third line, Levodopa/Carbidopa Intestinal Gel (LCIG) and Levodopa/Carbidopa/Entacapone Intestinal Gel (LECIG) can be considered. The same principles apply to patients without psychiatric comorbidities or cognitive deficits, who, however, due to comorbidities, have a significantly increased surgical risk, as indicated in Fig. 1.

In patients with pronounced postural instability, the indication for DBS should be approached with caution, and other DAT should be prioritized.

Patients seeking confirmation of the future effectiveness before the induction of a DAT can be offered all procedures except for DBS. However, it should be noted that the levodopa response serves as an indirect marker for a future DBS response, which has proven valuable in clinical practice.

Patients with a tremor that does not respond adequately to levodopa should be treated with DBS.

The presence of significant cognitive deficits or a pharmacorefractory psychosis argues against DBS or CSAI. However, in close collaboration between the patient’s caregivers and the therapeutic team, e.g. neurologists, psychiatrist, gastroenterologists, LCIG/LECIG or foslevodopa/foscarbidopa may be considered.

Regarding non-motor symptoms, the data are overall heterogeneous, particularly limited in comparability due to diverse patient populations and small sample sizes. However, when considered collectively, these study suggest that DAT can lead to a significant clinical improvement in several non-motor symptoms. In patients with ICBDs, DBS should be considered when an optimization of the oral medication does not result in appropriate symptom alleviation.

## Conclusion

In summary, LCIG/LECIG, foslevodopa/foscarbidopa, CSAI and DBS do not compete with each other, but rather complement each other. In order to maintain or improve quality of life and participation of patients in social life, the indication for initiating such therapy should be continuously examined and the selection of the individual therapy should be tailored to the symptoms in a shared decision manner with patients and caregivers. Pump-based procedures should not be used to prolong the time to DBS implantation in appropriate candidates since there is a clear age-associated increase in peri-interventional adverse events. On the other hand, pump-based therapies are often a beneficial therapy option for patients who are no longer eligible for deep brain stimulation due to contraindications such as cognitive deficits and multimorbidity or in patients who refuse DBS. In addition to the sequential use of these continuous therapies, the combination of DBS and pump-based methods can also be used in selected cases, contributing to the best possible care for patients.
